# Muscle prestimulation tunes velocity preflex in simulated perturbed hopping

**DOI:** 10.1038/s41598-023-31179-6

**Published:** 2023-03-20

**Authors:** Fabio Izzi, An Mo, Syn Schmitt, Alexander Badri-Spröwitz, Daniel F. B. Haeufle

**Affiliations:** 1 Hertie Institute for Clinical Brain Research and Center for Integrative Neuroscience, University of Tübingen, Tübingen, Germany; 2 Dynamic Locomotion Group, Max Planck Institute for Intelligent Systems, Stuttgart, Germany; 3 Institute for Modelling and Simulation of Biomechanical Systems, University of Stuttgart, Stuttgart, Germany; 4 Department of Mechanical Engineering, KU Leuven, Leuven, Belgium

**Keywords:** Computational models, Skeletal muscle

## Abstract

Muscle fibres possess unique visco-elastic properties, which generate a stabilising zero-delay response to unexpected perturbations. This instantaneous response—termed “preflex”—mitigates neuro-transmission delays, which are hazardous during fast locomotion due to the short stance duration. While the elastic contribution to preflexes has been studied extensively, the function of fibre viscosity due to the force–velocity relation remains unknown. In this study, we present a novel approach to isolate and quantify the preflex force produced by the force–velocity relation in musculo-skeletal computer simulations. We used our approach to analyse the muscle response to ground-level perturbations in simulated vertical hopping. Our analysis focused on the preflex-phase—the first 30 ms after impact—where neuronal delays render a controlled response impossible. We found that muscle force at impact and dissipated energy increase with perturbation height, helping reject the perturbations. However, the muscle fibres reject only 15% of step-down perturbation energy with constant stimulation. An open-loop rising stimulation, observed in locomotion experiments, amplified the regulatory effects of the muscle fibre’s force–velocity relation, resulting in 68% perturbation energy rejection. We conclude that open-loop neuronal tuning of muscle activity around impact allows for adequate feed-forward tuning of muscle fibre viscous capacity, facilitating energy adjustment to unexpected ground-level perturbations.

## Introduction

Muscles are smart actuators; they generate forces and movements and contribute to controlling them. Thanks to muscles’ nonlinear mechanical characteristics, they can react to unexpected perturbations instantly^1–5^ and without the inherent delays of neuronal reflexes^6^. This zero-delay capacity is known as “preflex”^7^. Preflexes allow mitigating neurotransmission delays, which otherwise produce significant reaction latencies (animal size dependent, > 30 ms*…*50 ms) and hinder the control of quick movements^5,6^.

**Figure 1:**
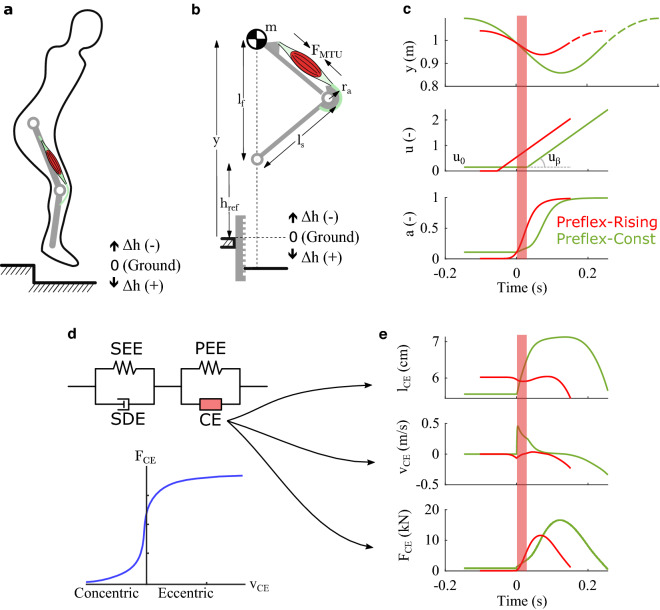
Study design. (**a**) Step perturbations present a challenge during agile locomotion because neuro-transmission delay affects a considerable fraction of the stance duration. Because of their instantaneous response to ground disturbances, the mechanical properties of muscle–tendon units may be critical to prevent falling. (**b**) We simulate vertical hopping of a massless, two-segment leg model to investigate the stabilising response of the muscle fibre’s force–velocity relation to step disturbance ∆*h*. Here, *h*_*ref*_ indicates the unperturbed periodic hopping height, and *y* is the vertical position of the point-mass* m.* Leg length during flight and leg segment length are indicated by *l*_*f*_ and* l*_*s*_, respectively*. F*_*MTU*_ is the force exerted by the muscle–tendon unit and* r*_*a*_ the associated lever arm. (**c**) To stimulate the muscle and generate periodic hopping, we apply a ramp signal, with prestimulation value *u*_0_ and slope *u*_*β*_. In Preflex-Const, the muscle stimulation starts at actual touch-down and stays constant (*u* = *u*_0_) until the end of the preflex duration (30 ms post touch-down, indicated by the colored bar, with *t* = 0 s being touch-down). In Preflex-Rising, the muscle stimulation starts before expected touch-down with *u*_0_ = 0. Here, the stimulation onset is fixed in time and thus independent of the actual touch-down timing. We use the activation dynamics in Refs.^24,25^ to convert the stimulation signal *u* into muscle activity *a*. We tuned Preflex-Const and Preflex-Rising to produce the same muscle activity at touch-down during unperturbed hopping: *y* shows the vertical trajectory of the center of mass for each stimulation strategy. Initial and final mass heights *y* are identical for each trajectory, indicating that both Preflex-Const and Preflex-Rising produced periodic hopping. (**d**) For our simulations, we use the Hill-type muscle model developed in Ref.^13^, which includes a contractile element (CE), a parallel elastic element (PEE), a serial elastic element (SEE), and a serial damper element (SDE). The force–velocity relation models the dependence of the muscle fibre force *F*_*CE*_ on the muscle fibre velocity *v*_*CE*_. Shortening velocities (negative *v*_*CE*_) define the concentric region of the force–velocity relation, while lengthening velocities (positive *v*_*CE*_) define the eccentric region. (**e**) Length (*l*_*CE*_), velocity (*v*_*CE*_) and force traces (*F*_*CE*_) of the muscle fibres during unperturbed hopping with Preflex-Const and Preflex-Rising.

Among the mechanical properties of muscle fibres is the capacity to produce higher muscle force generation when lengthening (eccentric contraction) than when shortening (concentric contraction)^8–11^. Macroscopic muscle models describe this asymmetry as a “force–velocity relation”^12,13^, i.e. a phenomenological relation between muscle fibre velocity and force (Fig. 1d).

Simulation studies have shown that the force–velocity relation can contribute to the stability of hopping and walking^1,14,15^. However, it is still unclear how the force–velocity relation contributes to the preflex mechanics. Previous research argued that the force–velocity relation implements a stabilising velocity preflex in muscle fibres^14,16^. If the impact velocity changes due to an unexpected ground height, the muscle’s force–velocity characteristics would instantly lead to an adjusted force (Fig. 1a). Similar self-regulating mechanisms are documented for parallel-elastic viscous damper systems during legged locomotion^17–19^.

Much functional research concerns the existence and shape of the force–velocity relation, and is often qualitative^14,15^. Multiple mechanisms rejecting ground perturbations are plausible; variables such as fibre length and neuronal activity shape the nonlinearity of muscle dynamics. For example, a ground perturbation during the rise of the muscle stimulation would trigger a change of muscle excitation at touch-down, leading to velocity-dependent regulation of muscle force similar to a velocity preflex. Furthermore, a change of impact velocity can also alter the muscle fibre stretch, which indirectly adapts muscle forces. Therefore, a quantitative analysis is necessary to test if the force–velocity relation leads to a velocity-dependent rejection of external disturbances, and if so, to what extent.

In this study, we use computer simulations to quantify velocity-dependent regulation of muscle fibre force when ground perturbations affect vertical hopping. The novelty of our analysis consists in the explicit quantification of the preflex response generated by the muscle fibres’ force–velocity relation, force–length relation, and neuronal activity. With this approach, we study the complex nonlinearity of muscle contraction, discerning the net contribution of the force–velocity relation from other regulating factors. Our results suggest that the force–velocity relation can regulate muscle forces during perturbed hopping in a velocity-dependent manner. However, we observe a substantial, stabilising response only if low-level neuronal stimulation interacts with the muscle’s force–length-velocity characteristics.

## Methods

### Musculoskeletal model

For our study, we used a modified version of the musculoskeletal model developed in Ref.^16^, with identical kinematics but a revised muscle–tendon unit^13^. The model consisted of a two-segment leg with total mass lumped at the hip and motion constrained to the vertical axis (Fig. 1b). During stance, the knee joint was actuated by the extensor muscle–tendon unit, whose force outcome *F*_*MTU*_ produced a torque equal to:

1 $$T_{knee} = r_{a} F_{MTU} ,$$

with *r*_*a*_ being the muscle lever arm. During the flight phase, the knee motion was always constrained and the leg length fixed to *l*_*f*_. This solution guaranteed the same leg geometry at touch-down despite the absence of antagonistic muscles in our model. Because of the simplified flight phase, we could reduce the analysis of each hopping scenario to the first hopping cycle (apex-to-apex). Each simulation started with the model in flight phase. Touch-down occurred when the mass vertical position *y* ≤ *l*_*f*_, and take-off when either *y* > *l*_*f*_ or the ground reaction force *F*_*leg*_ ≤ 0N (positive *F*_*leg*_ was upwards directed).

For our muscle–tendon unit, we used the Hill-type model in Ref.^13^. It consists of four elements, as shown in Fig. 1d: a contractile element (CE), a parallel elastic element (PEE), a serial elastic element (SEE), and a serial damping element (SDE). These four components fulfil the force equilibrium:

2 $$F_{MTU} = F_{CE} + F_{PEE} = F_{SEE} + F_{SDE} .$$

The contractile element represents the collective contribution of the muscle fibres to the muscle contraction. In our model, the parallel elastic element (PEE) engages when *l*_*CE*_ > 95% *l*_*opt*_, i.e. when the muscle fibre length (*l*_*CE*_) is close to the muscle fibre optimal length (*l*_*opt*_*)*. This condition never occurred in our simulations, which means that in our study, the force produced by the muscle fibres (*F*_*CE*_) was equal to the total force produced by the muscle–tendon unit (*F*_*MTU*_).

The force of the contractile element *F*_*CE*_ is a non-linear function of the fibre velocity *v*_*CE*_ (force–velocity relation), fibre length *l*_*CE*_ (force–length relation), and muscle activity *a*, the latter in turn dependent on the neuronal stimulation *u* received by the muscle fibres. The force–velocity relation comprises two regions, shown in Fig. 1d: the concentric and the eccentric contraction regions, characterised by shortening velocities (*v*_*CE*_ ≤ 0 m/s) and stretching velocities (*v*_*CE*_ > 0 m/s) of the muscle fibres, respectively. The force–velocity relation predicts a flattening profile of *F*_*CE*_ for increasing eccentric velocities. Such flattening is suggested by experimental data and commonly implemented in biomechanical models; for an overview see Fig. 3 of Ref.^20^.

We implemented our model in Simulink R2018a (Mathworks Inc., Natick, MA, USA). We used ode45 as numerical solver, with a maximum step size of 10^−4^, and absolute and relative error tolerances of 10^−8^. Model parameters are listed in Table1. A more in-depth list of model variables can be found in the electronic supplementary materials (Table S1). Note that our model requires a large value for the maximum isometric force *F*_*max*_ to compensate for the lack of additional muscle–tendon units, as explained in Ref.^16^.

**Table 1:** Model parameters, adapted from Ref^16^.

Parameter	Value
Body weight $m$	80 kg
Gravitational constant $g$	9.81 m/s^2^
Assumed flight leg length ${l}_{f}$	0.99 m
Segment length ${\mathcal{l}}_{s}$	0.5 m
Optimal muscle fibre length ${\mathcal{l}}_{opt}$	0.1 m
Lever arm ${r}_{a}$	0.04 m
Maximum isometric force ${F}_{max}$	22 kN
Activation dynamics time constant $\tau$	88.5 ms

### Muscle stimulation

This study focuses on the preflex phase, i.e. the first 30 ms after touch-down, during which neuronal feedback is absent because of neuronal delays^6^. To initialize hopping, muscle fibres receive a ramp signal as neuronal stimulus *u*(*t*) (Fig. 1c). The ramp signal is inspired by the observation that knee extensor’s activity rises during locomotion around and after impact^21–23^. We implemented two stimulation protocols: Preflex-Const (*u*_*C*_) and Preflex-Rising (*u*_*R*_).

With Preflex-Const, the ramp signal is kept constant during the first 30 ms after touch-down. After the preflex phase, the muscle stimulation rises linearly:

3 $${u}_{C}\left(t\right)=\left\{\begin{array}{*{20}l}{u}_{0}+{u}_{\beta }\left(t-{t}_{TD}-{\delta }_{C}\right), & \, {\text{for}} \, t>{t}_{TD}+{\delta }_{C}\\ {u}_{0}, & \, {\text{otherwise}} \, \end{array},\right.$$

where *u*_0_ is the muscle prestimulation level, *u*_*β*_ the slope of the ramp stimulus, *t*_*TD*_ the time of touch-down, and *δ*_*C*_ = 30 ms the neuronal delay. The Preflex-Const protocol enabled us to study the isolated response of the muscle fibres’ inherent, mechanical properties. The Preflex-Const protocol also served as design reference for Preflex-Rising.

With Preflex-Rising, the muscle stimulation *u*(*t*) rises linearly starting 54 ms before *expected* touch-down, with the same linear coefficient as in Preflex-Const (*u*_*β*_):

4 $${u}_{R}\left(t\right)=\left\{\begin{array}{*{20}l}{u}_{\beta }\left(t-{t}_{TD}^{*}+{\delta }_{R}\right), & \, {\text{for}} \, t>{t}_{TD}^{*}-{\delta }_{R}\\ 0,& \, {\text{o}}{\text{t}}{\text{h}}{\text{e}}{\text{r}}{\text{w}}{\text{i}}{\text{s}}{\text{e}} \, \end{array},\right.$$

where ${t}_{TD}^{*}$ is the *expected* touch-down event, i.e. the time in which touch-down occurs during unperturbed hopping, and *δ*_*R*_ = 54 ms. We applied the particular time offset *δ*_*R*_ to ensure that both Preflex-Rising and Preflex-Const produced unperturbed periodic hopping with the same amount of muscle activity at touch-down. Therefore, Preflex-Const and Preflex-Rising guarantee the same muscle pre-activation level at touch-down, as shown by the *a*-signal in Fig. 1c. However, while the onset of Preflex-Const depends on the *real* touch-down *t*_*TD*_, Preflex-Rising is an open-loop signal with the onset at the *expected* touch-down (${t}_{TD}^{*}$), and therefore independent of the actual ground height. The Preflex-Rising protocol allowed comparing Preflex-Const results with those of a more biologically feasible stimulation, for which inherent mechanics and feedforward control of muscle fibres interact to generate a first response to ground perturbations.

Regardless of the stimulation protocol in use, our model includes the activation dynamics described in Refs.^24,25^, which consists of a first order differential equation incorporating fibre length dependency. The activation dynamics turn the stimulation signal *u*(*t*) into muscular activity *a*(*t*) before reaching muscle fibres, limiting muscle activity between 0.005 and 1.

### Muscle fibre force decomposition

We aimed at quantifying the contribution of the force–velocity relation to the force produced by the muscle fibres during the preflex phase. We further separated the contributions of fibre elasticity and muscle activity. In our simulations, the muscle fibres exert a force

5 $$F_{CE} \left( t \right) \, = f_{CE} \left( {v_{CE} \left( t \right),l_{CE} \left( t \right),a\left( t \right)} \right),$$

which is a non-linear function of the muscle fibre velocity *v*_*CE*_(*t*), length *l*_*CE*_(*t*), and muscle activity *a*(*t*). For better readability, we will omit the time-dependency in the following.

We can separate the three contributions by interpreting *f*_*CE*_ as follows:

6 $${f}_{CE}={\int }_{{t}_{TD-1}}^{{t}_{x}}\frac{d{f}_{CE}\left({v}_{CE},{l}_{CE}, a\right)}{dt}dt+{F}_{CE}^{0},$$

where ${F}_{CE}^{0}$ is the force exerted by the muscle fibres immediately before touch-down, *t*_*TD-1*_ is the time instance just before touch-down and *t*_*x*_ any later time within the stance duration. After applying the chain rule in Eq. (6), we can rewrite Eq. (5) as the sum of four components:

7 $$\begin{array}{*{20}l} {F_{CE} = F_{CE}^{V} + F_{CE}^{L} + F_{CE}^{A} + F_{CE}^{0} } \hfill \\ {F_{CE}^{V} = \int\limits_{{t_{TD - 1} }}^{{t_{x} }} {\frac{{\partial f_{CE} }}{{\partial v_{CE} }}dv_{CE} } } \hfill \\ {F_{CE}^{L} = \int\limits_{{t_{TD - 1} }}^{{t_{x} }} {\frac{{\partial f_{CE} }}{{\partial l_{CE} }}dl_{CE} } } \hfill \\ {F_{CE}^{A} = \int\limits_{{t_{TD - 1} }}^{{t_{x} }} {\frac{{\partial f_{CE} }}{\partial a}da} } \hfill \\ \end{array} ,$$

where ${F}_{CE}^{V}$ is the force contribution of the force–velocity relation, ${F}_{CE}^{L}$ of the force–length relation, and ${F}_{CE}^{A}$ of the muscle activity, the latter in turn associated with the stimulation signal received by the muscle fibres.

We implemented this decomposition algorithm offline, using the trapezoidal rule to solve each integral in Eq. (7) numerically (*cumtrapz*, Matlab R2018a, Mathworks Inc., Natick, MA, USA). Touch-down (*t* = *t*_*TD*_) occurs instantly in our model, hence we computed the initial values of the numerical integration of Eq. (7) algebraically:

8 $$\begin{array}{c}{F}_{CE}^{L}\left({t}_{TD}\right)={F}_{CE}^{A}\left({t}_{TD}\right)=0\\ {F}_{CE}^{V}\left({t}_{TD}\right)={F}_{CE}\left({t}_{TD}\right)-{F}_{CE}^{0}\end{array},$$

Equation (8) shows that changes in muscle fibre force at touch-down must derive from the contribution of the force–velocity relation, since muscle fibre length (*l*_*CE*_) and activity (*a*) remain constant at the instantaneous ground impact.

### Hopping motion and perturbation modelling

To explore the contribution of muscle fibres to preflexes during perturbed hopping, we established reference hopping conditions for both stimulation protocols, i.e. Preflex-Const and Preflex-Rising.

We started by tuning the Preflex-Const stimulus to produce periodic hopping with physiologically-plausible muscle activation values: pre-stimulation value *a*_*0*_ = 0.11, reaching 90% saturation in 117 ms. Setting *u*_0_ = 0*.*15 and *u*_*β*_ = 10 resulted in vertical hopping with 10.6 cm apex height (*h*_*ref*_) and the muscle activity shown in Fig. 1c. The resulting stance duration was 255 ms and the hopping frequency 1.8 Hz, consistent with periodic hopping investigated by previous research^16^. Although the Preflex-Const stimulation *u*_*C*_ remained constant throughout the preflex phase, in accordance to Eq. (3), a small increase of muscle activity *a* occurred within the preflex duration (see bottom plot, green line in Fig. 1c). This increase is a result of the activation dynamics’ dependency on the muscle fibre length and stretch throughout the preflex duration.

We designed the Preflex-Rising stimulation using Preflex-Const as reference: we set *δ*_*R*_ = 54 ms to have the same amount of muscle activation *a* at touch-down (*a*_0_ = 0.11), and *u*_*β*_ = 10 for an equal rate of muscle stimulation—producing 90% saturation of *a*-signal in 70 ms after touch-down. These settings resulted in a Preflex-Rising muscle activity comparable to that produced by Preflex-Const, as shown in Fig. 1c. The major difference is a time shift due to the lack of a constant stimulation phase during the preflex. The Preflex-Rising stimulation produced periodic hopping with an apex height *h*_*ref*_ of 5.2 cm, a stance duration of 151 ms, and a hopping frequency of 2.8 Hz. These features imply that Preflex-Rising caused stiffer reference hopping than Preflex-Const, as also seen by the smaller change in the *l*_*CE*_ and *v*_*CE*_ time traces in Fig. 1e. Nonetheless, the characteristics of the stiffer reference periodic hopping remains in line with earlier studies^16^.

A second difference in the periodic hopping associated with Preflex-Rising and Preflex-Const was the muscle fibre’s force at touch-down: 179N during Preflex-Rising, and 1260N during Preflex-Const. The difference in touch-down conditions occurs as the muscle stimulation starts before touch-down in the periodic hopping caused by Preflex-Rising. As a result, muscle fibres are shortening at ground contact with Preflex-Rising, and this causes the concentric side of the force–velocity relation to diminish the muscle fibre force at touch-down. Figure 1e further illustrates the time trace of *F*_*CE*_ for both stimulation protocols.

After selecting these reference hopping conditions for the two investigated stimulation protocols, we simulated unexpected ground perturbations by applying changes ∆*h* to the reference hopping height. We applied a total of six height perturbations, consisting of two step-up (∆*h* =  − 5.0 cm and − 2.5 cm) and four step-down perturbations (∆*h* = 2.5 cm, 5.0 cm, 7.5 cm and 10.0 cm). Hence, ground perturbations ranged from − 5 to 10%, in reference to a leg length of *l*_*f*_ = 99 cm. Limiting the largest step-up perturbation to − 5 cm was because *h*_*ref*_ = 5.2 cm during reference periodic hopping with Preflex-Rising.

**Figure 2:**
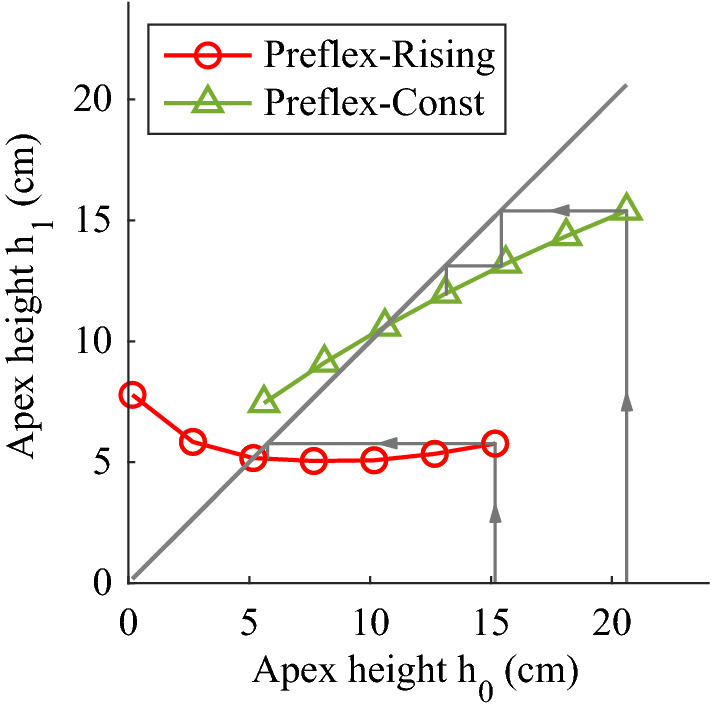
Apex return map for Preflex-Const (green) and Preflex-Rising (red). *h*_0_ is each initial drop height tested in our study, and *h*_1_ each associated return apex after one hopping cycle. Notice that *h*_1_ would be the initial drop height for the following hopping cycle during continuous hopping. Intersections with the diagonal line represent periodic hopping heights. The rather horizontal return-map for Preflex-Rising around its periodic hopping height represents faster rejection of ground perturbations: the arrows indicate the faster convergence to periodic hopping within fewer hopping cycles.

The apex-return map visualizes the stability of Preflex-Const and Preflex-Rising reference hopping against the tested step perturbations (Fig. 2). The apex-return map plots each tested drop height (*h*_0_ = *y(t*_*TD*_*) − l*_*f*_) against the apex height at the end of the first hopping cycle (*h*_1_)—which would become the follow up drop cycle height. A horizontal apex-return profile would show complete rejection of any ground disturbance since any perturbation intensity will produce a return apex coincident with the reference hopping height. If the apex-return profile coincides with the diagonal line, the result is periodic hopping at the perturbed hopping height. The hopping height would not convergence back to the initial, reference hopping height, and the perturbation would not be rejected. Finally, any apex-return profile with a slope outside the area enclosed by (1) the diagonal line and (2) its perpendicular line at the periodic hopping height represents unstable hopping: following a ground perturbation, the return apex will move away from the reference hopping height at each new hopping cycle.

Figure 2 shows that reference hopping of Preflex-Const and Preflex-Rising were asymptotically stable for the set of step perturbations studied here. Preflex-Rising showed faster rejection of the step-down perturbations, based on its more horizontal apex-return profile in Fig. 2.

## Results

### Touch-down response (at t = 0 ms)

**Figure 3:**
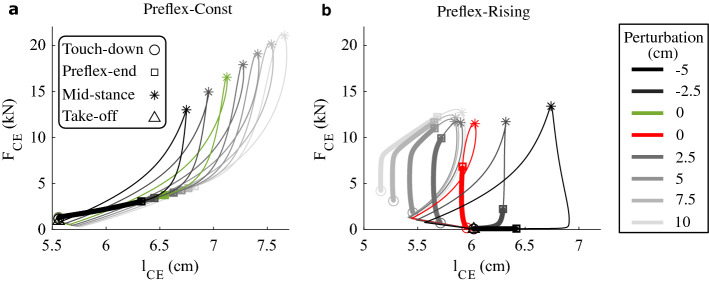
Muscle fibre workloop results for (**a**) Preflex-Const and (**b**) Preflex-Rising. Data is plotted from touch-down (open circle) to take-off (open triangle). The preflex phase is emphasised with thicker lines. Open squares indicate the end of the preflex duration, asterisks indicate mid-stance. The drop height increases from darker to lighter colors: the darkest and lightest workloops are produced by the largest step-up and step-down perturbations, respectively. Reference hopping conditions are coloured green and red for Preflex-Const and Preflex-Rising, respectively.

**Figure 4:**
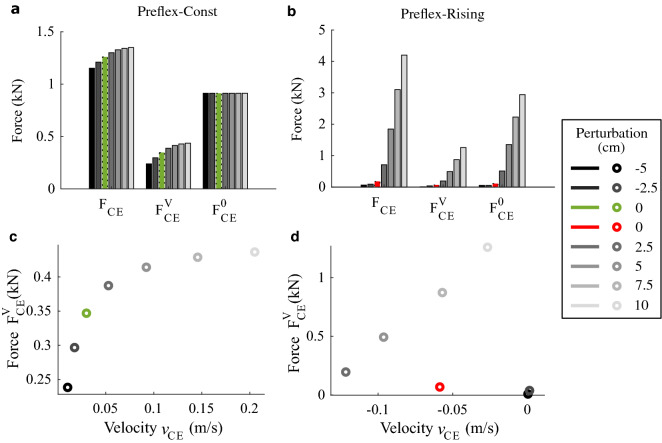
Touch-down results. (**a,b**) Force values: *F*_*CE*_ is the net muscle fibre force, ${F}_{CE}^{V}$ the force component due to the force–velocity relation and ${F}_{CE}^{0}$ the muscle fibre force just before touch-down. (**c,d**) Touch-down dependence of the muscle fibre force component due to the force–velocity relation (${F}_{CE}^{V}$) on the muscle fibre velocity (*v*_*CE*_). (**a,c**) Results for Preflex-Const, with reference hopping condition in green. (**b,d**) Results for Preflex-Rising, with reference hopping condition in red.

At touch-down, Preflex-Const produced little adjustments of muscle fibres’ force in response to ground perturbations, while Preflex-Rising produced larger adjustments. This finding is visible at the touch-down events in Fig. 3, indicated by circle symbols. Figure 4a shows that during Preflex-Const, the muscle fibre force *F*_*CE*_ ranged from 1.15 to 1.35 kN at impact. Also, changes in *F*_*CE*_ became smaller as the drop height increased. During Preflex-Rising, *F*_*CE*_ at impact displayed a wider range of adjustment (from 0.06 to 4.20 kN, Fig. 4b). Here, the majority of change occurred along the step-down perturbations. This finding is expected since with Preflex-Rising, the concomitant effects of delayed ground impacts and rising stimulation produce larger muscle excitation at touch-down during step-down perturbations.

By separating the individual components contributing to the muscle force at touch-down, it becomes clear that different mechanisms lead to the touch-down adjustment of *F*_*CE*_ (Fig. 4a,b), depending on the stimulation protocol. During Preflex-Const, the force–velocity relation was the main factor driving *F*_*CE*_ adjustment, as shown by changes in ${F}_{CE}^{V}$ and constant values of ${F}_{CE}^{0}$ across the tested conditions (Fig. 4a). During Preflex-Rising, changes in both ${F}_{CE}^{V}$ and ${F}_{CE}^{0}$ produced *F*_*CE*_ adjustment, with ${F}_{CE}^{0}$ contributing the most (Fig. 4b). This shows that the adjustment of muscle stimulation at touch-down, which occurs during Preflex-Rising but not during Preflex-Const, plays a major role in adjusting the initial preflex response to the perturbation intensity.

We hypothesised that the force–velocity relation contributes to regulate ground perturbations by means of a velocity preflex. Such a velocity-dependence can be seen when plotting the force component ${F}_{CE}^{V}$ against muscle fibre velocities *v*_*CE*_ at impact (Fig. 4c,d). The plot reveals that the touch-down event of Preflex-Const trials always occurred in the eccentric contraction, and that increasing drop heights led to faster *v*_*CE*_ but flatter adjustment of ${F}_{CE}^{V}$. The flattening of ${F}_{CE}^{V}$ adjustment is a result of the eccentric side of the force–velocity relation in our muscle model, which predicts a decrease in velocity-produced adjustment of the muscle force as *v*_*CE*_ increases (Fig. 1d). No flattening adjustment of ${F}_{CE}^{V}$ occurred during Preflex-Rising trials. In contrast, the rising muscle stimulation caused an almost linear trend along step-down perturbations. Furthermore, during Preflex-Rising trials, touch-down occurred in concentric contraction. Exceptions were the two step-up perturbations, which produced touch-down with almost steady conditions (|*v*_*CE*_|< 0.01 m/s) and minimal muscle activity due to the short time for the muscle stimulation to rise.

### Preflex response (t = 0 ms to 30 ms)

Workloop trajectories of Preflex-Const trials were almost identical during preflex (*t* = 0 ms to 30 ms). They mainly differed by the amount of stretch reached by muscle fibres at the end of preflex, shown by the box symbols in Fig. 3a shifting to the right. Hence, differences in the muscle fibre velocity between trials resulted in minimal adjustment of the muscle fibre force throughout the preflex duration. Therefore, in Preflex-Const trials, increased energy dissipation during preflex was mainly caused by larger fibre stretch.

In contrast, workloop trajectories of Preflex-Rising trials show more complex preflex adjustments, visible by the variety of workloop shapes (Fig. 3b). Step-up perturbations of higher intensity resulted in more eccentric stretch of muscle fibres and lower rise in muscle fibre force during preflex, as shown by the two darker lines in Fig. 3b. This trend emerges because larger step-up perturbations move the touch-down event closer to the onset of muscle stimulation. This leads to less excited and, therefore more compliant, muscle fibres during preflex. For instance, the − 5.0 cm step-up perturbation shifted the touchdown event about 30 ms before the muscle stimulation onset, making the muscle fibres relaxed for 99% of the preflex phase and dissipate almost no energy. The step-down perturbations consisted of an initial phase of concentric shortening, followed by an eccentric stretch of the muscle fibres. Larger perturbations caused more prominent eccentric phases with larger muscle fibre forces, resulting in more energy dissipated by muscle fibres during preflex.

**Figure 5:**
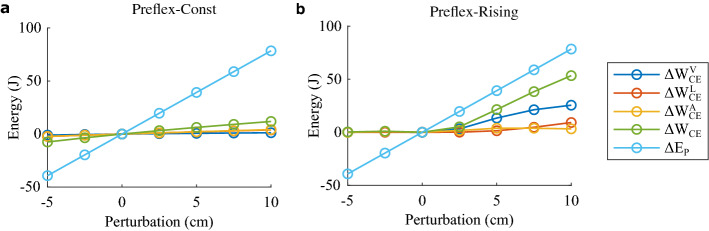
Change in muscle fibre work caused by each step perturbation (circles) with respect to the reference hopping condition. ∆*E*_*P*_ (light blue) is the amount of work adjustment required to reject, within the preflex duration, the potential energy change caused by each step perturbation. ∆*W*_*CE*_ (green) is the net change in muscle fibre work. ∆${W}_{CE}^{V}$ (dark blue) is the change in muscle fibre work induced by the force–velocity relation; ∆${W}_{CE}^{L}$ (red) by the force–length relation; ∆${W}_{CE}^{A}$ (yellow) by the muscle excitation. (**a**) Preflex-Const and (**b**) Preflex-Rising.

To investigate the amount of energy regulation, we calculated the change of muscle fibre work during preflex in response to each ground perturbation (∆*W*_*CE*_, green lines in Fig. 5). The light-blue line (∆*E*_*P*_ in Fig. 5) indicates the amount of potential energy altered by each step perturbation. As such, ∆*W*_*CE*_ matching ∆*E*_*P*_ indicates the full rejection of ground perturbations by the end of the preflex phase.

Results show that Preflex-Rising produced a consistently better regulation of muscle fibre work than Preflex-Const during step-down perturbations. For example, during the largest step-down perturbation, ∆*W*_*CE*_ = 53.4 J with Preflex-Rising, and ∆*W*_*CE*_ = 11.9 J with Preflex-Const. This means that Preflex-Rising could reject 68% ∆*E*_*P*_, while Preflex-Const only 15% ∆*E*_*P*_. Preflex-Const adapted well to step-up perturbations by reducing muscle fibre work (negative x-axis, Fig. 5a). During the largest step-up perturbation, ∆*W*_*CE*_ =  − 7.5 J with Preflex-Const, meaning 19% ∆*E*_*P*_ rejection. In contrast, Preflex-Rising failed to adjust muscle fibre work during step-up perturbations (negative x-axis, Fig. 5b). Instead, muscle fibre work slightly increased: ∆*W*_*CE*_ was equal to 1.0 J and 0.3 J for − 2.5 cm and − 5.0 cm step-up perturbations, respectively.

We hypothesised that the force–velocity relation is important to regulate the muscle fibre work in response to perturbed ground contacts. We were able to test our hypothesis with our new approach separating the components that produce ∆*W*_*CE*_. We identified different principles of work regulation between Preflex-Const and Preflex-Rising. During Preflex-Const, we found a similar contribution from the force–velocity relation (∆${W}_{CE}^{V}$, dark-blue line), force–length relation (∆${W}_{CE}^{L}$, red line), and muscle excitation (∆${W}_{CE}^{A}$, yellow line). In particular, ∆${W}_{CE}^{V}$ contributed the least to ∆*W*_*CE*_ during Preflex-Const trials. In contrast, during Preflex-Rising ∆${W}_{CE}^{V}$ contribution was predominant over ∆${W}_{CE}^{L}$ and ∆${W}_{CE}^{A}$. These results show that by adjusting muscle prestimulation at touch-down, the regulatory effect of the force–velocity relation on the muscle fibre work is most effective.

## Discussion

In this study, we tested whether muscle fibres can produce velocity preflex, i.e. a mechanical velocity feedback, to reject step perturbations, and how much the force–velocity relation contributes. We tested two stimulation protocols; in Preflex-Const, the stimulation of muscle fibres is kept constant around impact as an artificial measure to isolate the mechanical contribution of the muscle fibres to preflex. The more physiologically plausible Preflex-Rising case permits to study the interplay between low-level control and muscle fibres mechanical properties. Thanks to our suggested analytical approach to quantify force components produced by muscle fibre mechanical properties, we found that the force–velocity relation does not produce a substantial velocity preflex in isolation, i.e., it produces only a minor adjustment of muscle fibre’s force to variable impact velocity. However, its regulating effect can be maximised by feedforward control.

### Reflection on literature hypothesis

By completely removing the force–velocity relation, previous research showed that the force–velocity relation is crucial to reject dynamic perturbations^1,14,15^. It was suggested that the force–velocity relation stabilises locomotion by providing damping capacities to the muscle fibres^14,16,26^. According to this hypothesis, in the event of a sudden step perturbation, the force–velocity relation would adjust the muscle fibre force in response to the new impact velocity. With this velocity preflex, that is, a mechanical velocity feedback embedded in the muscle fibres, the force–velocity relation can mitigate delays caused by neuro-transmission, and reduce feedback control by the nervous system.

By explicitly quantifying the preflex response of muscle fibres during perturbed hopping with constant muscle stimulation around impact (Preflex-Const), we observed that the muscle fibre force does, indeed, adjust to the perturbed impact velocity because of the force–velocity relation (Fig. 4a) as previously hypothesised. However, this regulatory action of the force–velocity relation was small and quickly faded (< 5 ms) during preflex (Fig. S1a, in Supplementary Material). As a result, the force–velocity relation produced minimal adjustment of the muscle work and had little effect on rejecting the potential energy induced by each step perturbation (Fig. 5a). One aspect which seems to limit the contribution of the force–velocity relation is its flattening shape along the eccentric side (Fig. 1d). It predicts a diminishing influence on the muscle fibre force for sufficiently large muscle fibre velocities. Inspection of the time traces (Figs. S1, S2, in supplementary material) confirms that shortly after touch-down, a rapid increase in the stretching velocity of muscle fibres occurs, leading to a convergence of multiple ${F}_{CE}^{V}$ trajectories. This convergence explains why workloop trajectories of Preflex-Const trials were mostly aligned during the preflex duration (Fig. 3a).

Our Preflex-Const tests reveal a limited energy regulation by the mechanical damping action associated to the force–velocity relation. Still, some energetic adaptation is achieved (Fig. 5a), but this is rather a result of the change in muscle fibre stretch during preflex, as can be seen from the area under the thick lines in the workloops (Fig. 3a).

Overall, our simulation results of Preflex-Const support the literature hypothesis about a velocity preflex induced by the force–velocity relation in musculoskeletal models. We also demonstrate its inefficacy in regulating ground perturbations in a purely passive mechanical manner. In contrast, the force–velocity relation becomes the predominant element in regulating perturbed hopping with Preflex-Rising. Here, a minimalist, low-level stimulation was sufficient to enhance the regulating action of the force–velocity relation, resulting in surprisingly complex stabilizing mechanics.

### Details about perturbation rejection by Preflex-Rising

Preflex-Rising produced better regulation of step-down perturbations. Its central aspect is the rising stimulation that amplified regulatory effects of the force–velocity relation (Fig. 5b). In human locomotion such rising stimulation can be found in knee extensor muscles^21–23^. Several previous computer simulations applied such control when investigating the role of the force–velocity relation or stability of gait^1,14,15,27,28^.

The underlying regulatory mechanism generated by Preflex-Rising shows an intricate interaction between muscle fibre force and velocity profile. The central effect is that increasing drop heights postponed ground impact and therefore lead to more excited muscle fibres at touch-down. On the one hand, the muscle is stiffer with rising activity and therefore resists shifting to eccentric velocities, where more energy can be dissipated. On the other hand, the rising activity consistently increased the force of the muscle fibre at impact with larger step-down perturbations. Furthermore, with more excited muscle fibres, the force–velocity relation scales vertically so that the change in muscle fibre velocity produces a larger response from the force–velocity relation, as shown in Fig. 4d. This scaling effect was already observed in Ref.^2^. Combined, these effects result in a substantial adaptation of the preflex energy dissipation to the perturbation height (Fig. 5b).

Now the intricate details: Preflex-Rising initiated a muscle contraction before impact, which led to an initial concentric contraction and then eccentric stretch during the preflex duration (Fig. S2b, in Supplementary Material). With increasing step-down perturbations, this initial contraction starts earlier and, depending on the timing of the perturbation, it shifts the shortening velocity of the fibres at impact (Fig. S2b, in Supplementary Material). For medium perturbations the contraction velocity was larger than in reference hopping. For large perturbations it was smaller, as muscle–tendon forces approached equilibrium (the knee is fixed in flight). In all cases, the ground impact causes a shift in muscle fibre velocity towards eccentric velocities. In particular, larger step-down perturbations produced a faster transition to the eccentric mode, and higher maximum eccentric velocity during preflex (Fig. S2b, in Supplementary Material). During Preflex-Rising, the dynamic effect of the step-down perturbation, which caused more eccentric stretch of the muscle fibres due to larger impact velocities, overcame the muscle fibre stiffening caused by the increasing muscle activity at touch-down. In combination with the faster rising of ${F}_{CE}^{V}$ due to the scaling of the force–velocity relation (Fig. S1b, in Supplementary Material), this specific trend in velocity profile resulted in muscular power generation that rose earlier, faster, and created a larger area during the preflex phase with increasing step-down height (Fig. S3b, in Supplementary Material).

It is worth noticing that the intricate details of how knee joint flexion is transmitted into muscle fibre stretch velocity depends on the internal stiffness of muscle fibres and tendons. At landing, changing the tendon’s stiffness affects both the way the impact velocity is transmitted to the muscle fibres and the overall stiffness of the muscle–tendon unit. These two aspects affect *v*_*CE*_ in opposite ways and can (potentially) balance each other. From one side, more compliant tendons will decouple the muscle fibres from the joint action^29^, thus favoring reduced *v*_*CE*_ values just after landing. However, the muscle–tendon unit will be more compliant overall, meaning that reference hopping will occur at higher hopping heights due to more elastic recoil during the stance phase. This increased hopping height favors higher impact velocities, and as a consequence, higher *v*_*CE*_ values.

Changing the tendon’s stiffness also alters how much *v*_*CE*_ changes following a step perturbation. Stiffer tendons will comport larger changes in *v*_*CE*_. Notice, however, that the force–velocity relation has a plateau on its eccentric side (Fig. 1d). For this reason, larger increments in *v*_*CE*_ comport minor adjustment of *F*_*CE*_ after a certain range of *v*_*CE*_ values is reached (Fig. 4c shows that this reduced *F*_*CE*_ adaptation occurs already for *v*_*CE*_ = 0.1 m/s).

Therefore, we expect that a different compliance of the tendon affects the hopping pattern of our model and the operative point on the force–velocity relation at touch-down, and thus the magnitude of the force–velocity contribution. However, we do not expect tendon compliance to affect our main finding, i.e., that the force–velocity relation contributes to a velocity preflex in the presence of low-level feedforward stimulation. This is because feedforward stimulation will always contribute to scale the force–velocity relation, regardless of the force–velocity state at landing and during the preflex duration. As a future research direction, we propose to quantify the force–velocity contribution over a range of different muscle fiber to tendon length ratios^30^, which could give insights into the role of different muscle–tendon unit morphologies in locomotion.

### Muscle model considerations

The muscle model used here is a variant of a macroscopic Hill-type muscle model described in Ref.^13^. Originally, Archibal Hill developed a mathematical formulation to fit his experimental data on frog muscle fibre contraction^31^. Later models included explicit elements, i.e. mathematical formulations, for the tendon and connective tissue parts too. Several different arrangements of such mechanical elements exist, all being in series or parallel to the fibre formulation. The model used here additionally includes an explicit damper element (SDE) in series to the contractile element (CE). This Hill-type muscle model with added SDE fits the biological data for all muscle contraction experiments better than muscle models without added serial damper^32^. Additionally, the added SDE allows to observe an impact velocity distribution between the fibre and the tendon part. A purely elastic tendon formulation, as used in other variants of Hill-type models, does not provide such capability. Consequently, the Hill-type model with an added damper in series has shown great accuracy when modelling dynamic motions. Similar to the parallel damping element in Ref.^33^, the serial damper element in Ref.^13^ is intended to capture the macroscopic effects of viscosity in muscle–tendon units, but in series to the fibre. It is, therefore, ideally suited for the analyses of such a contribution on a macroscopic level.

The Hill-type muscle model used in this study also replicates the flattening of the force–velocity relation at high fibre-stretching velocities, which is consistent with previous experimental research on eccentric contraction of the muscle fibres^11^. However, the data on eccentric contractions is relatively sparse and the real dynamics more complicated, especially its inter-dependency with the force–length relation^34^. Nonetheless, our—or similar—implementations of the force–velocity relation allows to simulate realistic locomotion patterns^1,26^, which are further used to investigate perturbations in locomotion^15,28,35–37^.

Generally, Hill-type muscle models are macroscopic, phenomenological approximations of biological muscle–tendon complexes. As such, they present several short-comings when compared to experimental data of muscle fibres’ contraction. For example, experimental studies show that parameters for Hill-type models, which are usually obtained from maximally activated muscles, adjust based on complex relations with the activation level^38^. For this reason, Hill-type models are less accurate for simulating muscle force with variable excitation level, as occurring in our study. Over the last years, attempts have been made to derive the macroscopic formulation of muscle–tendon dynamics from a microscopic, biophysical perspective^39,40^. Thus, the macroscopic force–velocity relation, as used here, becomes the result of a first principles ansatz and a stringent derivation using few additional assumptions^40^. Looking inside biophysical models reveals explicit formulations of damping components inside the CE. Such formulations would facilitate the research presented here by enabling to directly quantify the damping rate at the muscle fibre level. Unfortunately, the existing models which incorporate explicit damping cannot yet predict the mechanical response of muscles during eccentric contractions^39,40^. This drawback leaves these more physiological-biophysical models currently insufficient to apply here, as the eccentric contraction is the main working mode during preflex.

### Implication of our results

Our study shows that the force–velocity relation contributes with a velocity preflex to reject unexpected ground perturbations, but it needs low-level control for maximum efficacy. This is consistent with Ref.^2^, where it was observed that time-based muscle stimulation drives the regulating response of muscle fibre viscoelasticity to variations in ground stiffness. In our simulation, the basic rising stimulation considered in Preflex-Rising was sufficient to amplify the regulatory effects of the force–velocity relation. However, even more simple stimulation strategies might produce similar effects. For example, Preflex-Const with prestimulation level proportional to the drop height, that is, with tunable *u*_0_ (see Eq. (3)), will produce the same vertical scaling of the force–velocity relation observed in Preflex-Rising, potentially leading to similar stabilizing effects. From a biological perspective, our findings raise the question of how much morphological computation, as described in Refs.^41,42^, is carried by the force–velocity relation, and whether a trade-off exists between the force–velocity relation’s stabilizing effect and control effort. From a technical perspective, understanding low-level control of the force–velocity relation can promote bio-inspired designs for assistive robotic devices, as numerous studies have suggested that implementing (tunable) dampers can improve the system performance and facilitate robustness against external perturbations^17,43^.

Previous research has qualitatively described a velocity preflex produced by the muscle fibre’s force–velocity relation to regulate locomotion^14–16^. With our quantitative analysis, which was made possible by our decomposition algorithm (see “Muscle fibre force decomposition” in “Methods” section), we could test the mechanical principles expected to produce such a velocity-based regulation by preflex. Our study shows that preflex regulation of perturbed hopping emerges from a complex coupling between muscle fibre stretching trajectories and muscle fibre force. The amount of preflex force produced by muscle fibres depends on the muscle fibre length-velocity state, which is in turn affected by the action of the preflex force on the knee joint flexion. In addition to low-level control, this intricate interaction is likely affected by several elements, such as ground impact dynamics, terrain stiffness, leg geometry and internal stiffness of the muscle–tendon unit. Therefore, future studies could consider more comprehensive musculoskeletal models to unveil the functionality of muscular preflex, and to allow direct comparison with experimental studies such as the running investigation carried out in Ref.^44^. In this context, the decomposition algorithm that we present here can become a central tool, as it permits to explicitly quantify the force components associated with muscular preflex despite of the model’s complexity.

## Supplementary Information

Supplementary Information.

## Data Availability

The Matlab/Simulink files needed to generate all the manuscript’s Results and Figures are available in Zenodo (10.5281/zenodo.7737860).
