# Supplementary material for: Muscle prestimulation tunes velocity preflex in simulated perturbed hopping
Source: Sci Rep. 2023 Mar 20;13:4559. doi: 10.1038/s41598-023-31179-6 (PMC10027857; doi:10.1038/s41598-023-31179-6)
Supplement: Supplementary file 1 — Supplementary Information. [file 41598_2023_31179_MOESM1_ESM.pdf]

Electronic supplementary material

Muscle prestimulation tunes velocity preflex in simulated perturbed hopping

Fabio Izzi[1,2,*], An Mo[2], Syn Schmitt[3], Alexander Badri-Spröwitz[2,4], Daniel F. B. Haeufle[1,3]

[1]Hertie Institute for Clinical Brain Research and Center for Integrative Neuroscience, University of Tübingen, Tübingen, Germany
[2]Dynamic Locomotion Group, Max Planck Institute for Intelligent Systems, Stuttgart, Germany
[3]Institute for Modelling and Simulation of Biomechanical Systems, University of Stuttgart, Stuttgart, Germany
[4]Department of Mechanical Engineering, KU Leuven, Leuven, Belgium
*Corresponding author's address: izzi@is.mpg.de

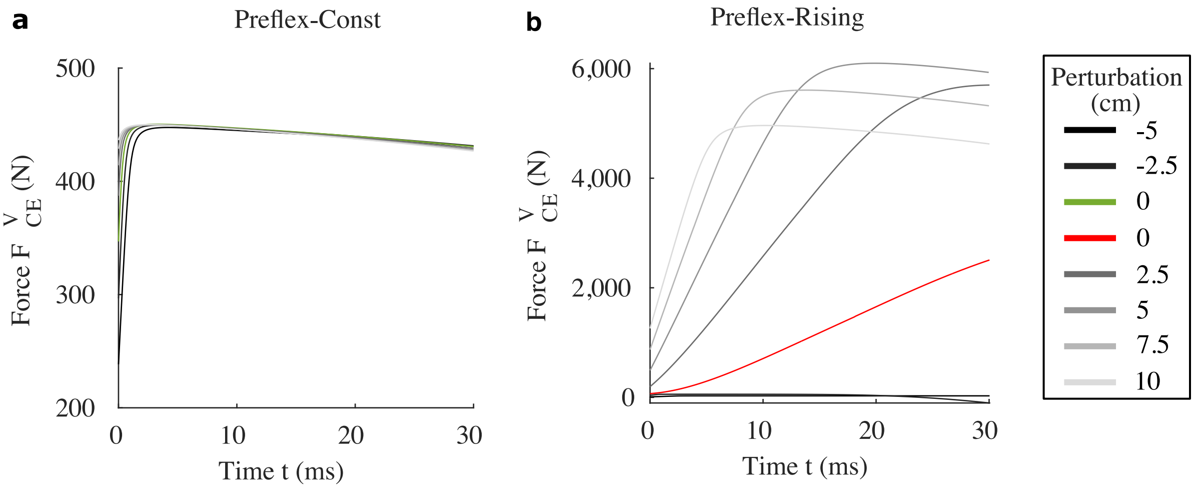

Fig. S1: Time trajectories of the force component produced by the force-velocity relation (F_{CE}^{V}). Data plotted from touch-down ($t = 0ms$) to the end of the preflex duration ($t = 30ms$). (a) Preflex-Const, with reference hopping case in green; (b) Preflex-Rising, with reference hopping case in red.

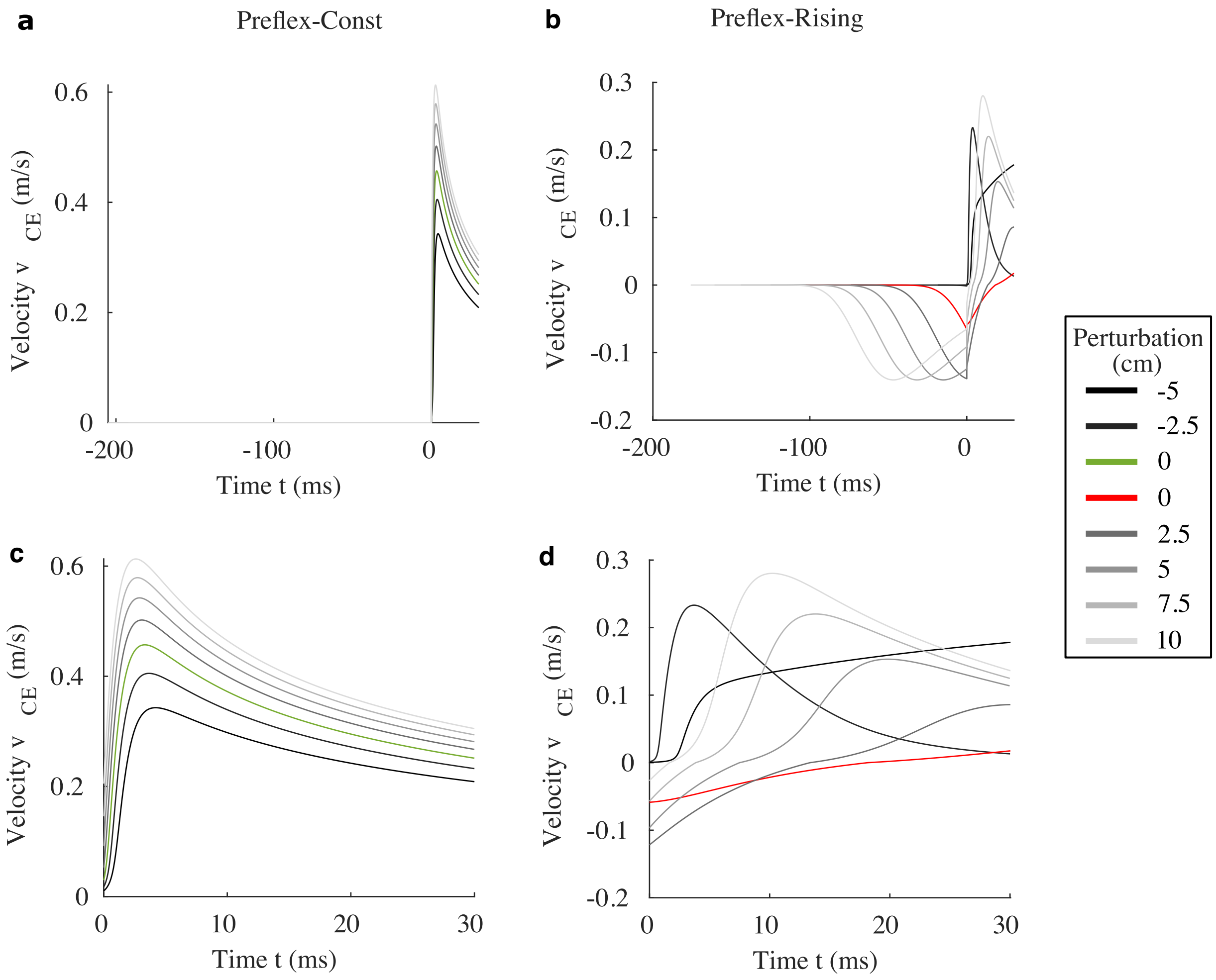

Fig. S2: (a,b) Time trajectories of the muscle fibre velocity (v_{CE}). Data plotted from the start of the leg's vertical fall to the end of the preflex duration ($t = 30ms$). All dataset are centered to the touch-down event ($t = 0ms$). (c,d) Close up (touch-down to preflex end) of the time trajectories of the muscle fibre velocity (v_{CE}). (a,c) Preflex-Const, with reference hopping case in green; (b,d) Preflex-Rising, with reference hopping case in red.

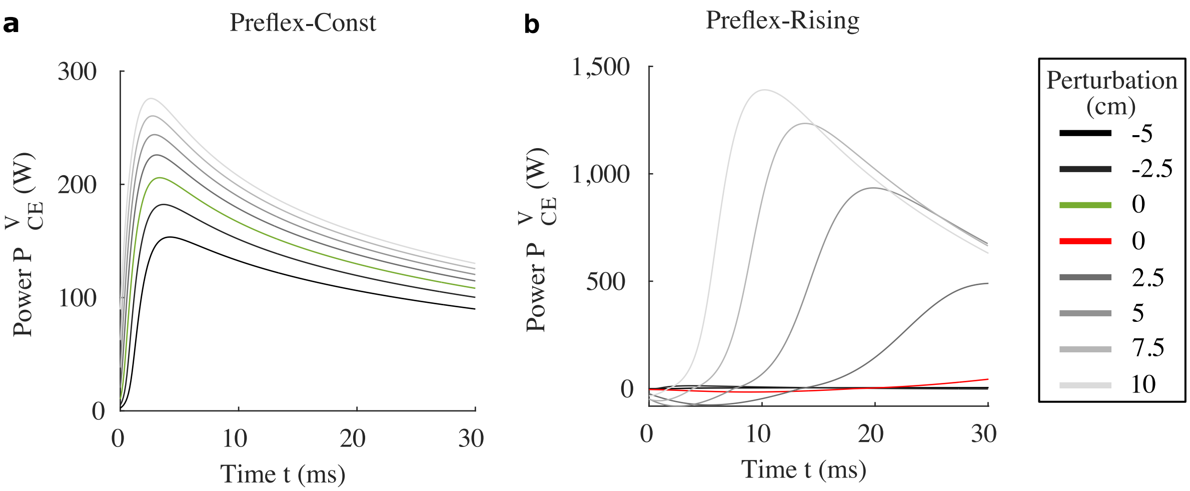

Fig. S3: Time trajectories of the muscle fibre power component produced by the force-velocity relation ($P^V_{CE} = F^V_{CE} \cdot v_{CE}$). Data plotted from touch-down ($t = 0ms$) to the end of the preflex duration ($t = 30ms$). (a) Preflex-Const, with reference hopping case in green; (b) Preflex-Rising, with reference hopping case in red.

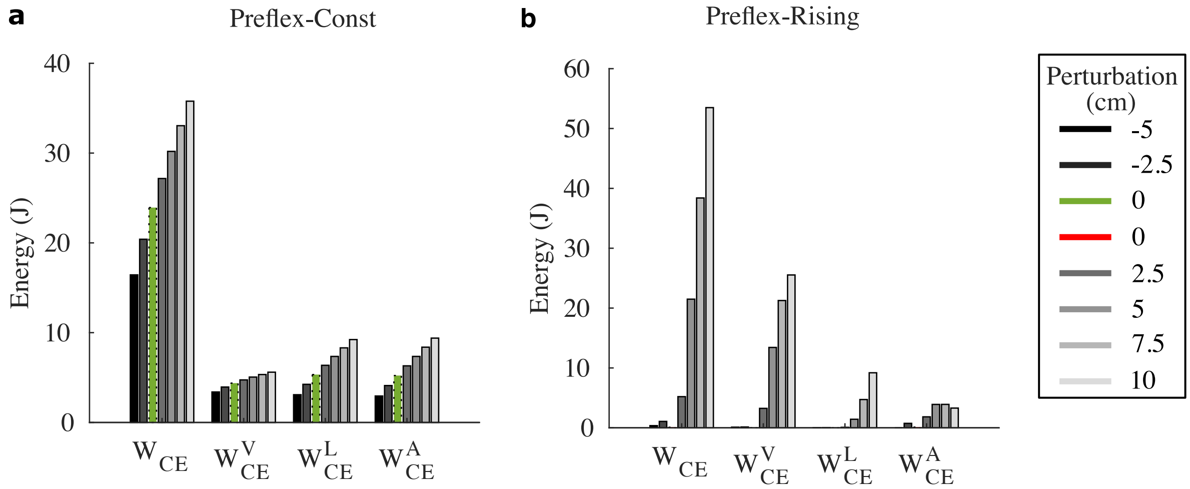

Fig. S4: Work dissipated by the muscle fibres during the preflex phase. W_{CE} is the net dissipated work; W_{CE}^{V} is the work component dissipated by the force-velocity relation, W_{CE}^{L} by the force-length relation, and W_{CE}^{A} by the muscle activity. (a) Preflex-Const, with reference hopping case in green; (b) Preflex-Rising, with reference hopping case in red.

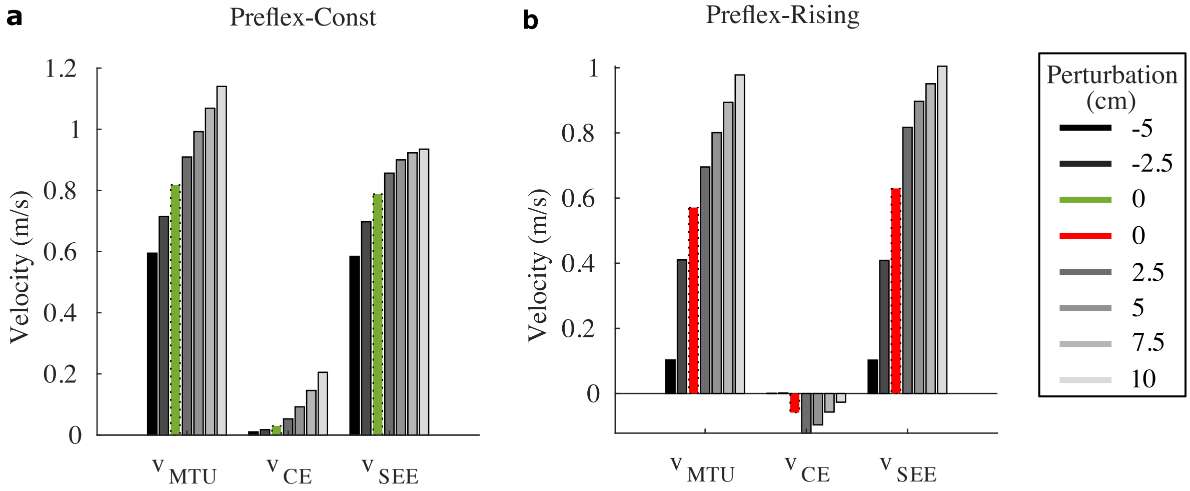

Fig. S5: Touch-down values of muscle-tendon unit velocity (v_{MTU}), muscle fibre velocity (v_{CE}) and tendon fibre velocity (v_{SEE}). (a) Preflex-Const, with reference hopping case in green; (b) Preflex-Rising, with reference hopping case in red.

TABLE S1: Table of supplementary parameters used in the muscle model and activation dynamics (Hatze).

	Parameter	Unit	Value	Source	Description
MTU	$l_{MTU,ref}$	m	0.5	Geyer et al. (2003)	muscle-tendon unit's reference length, alias l_{ref} in Geyer et al. (2003)
CE	ΔW^{des}	[ ]	0.45	similar to Bayer et al. (2017); Kistemaker et al. (2006)	width of normalized bell curve in descending branch, adapted to match observed force-length curves
	ΔW^{asc}	[ ]	0.45	similar to Bayer et al. (2017); Kistemaker et al. (2006)	width of normalized bell curve in ascending branch, adapted to match observed force-length curves
	$\nu_{CE,des}$	[ ]	1.5	Mörl et al. (2012)	exponent for descending branch of force-length relation
	$\nu_{CE,asc}$	[ ]	3.0	Mörl et al. (2012)	exponent for ascending branch of force-length relation
	$A_{rel,0}$	[ ]	0.2	Bayer et al. (2017)	parameter for contraction dynamics: maximum value of A_{rel}
	$B_{rel,0}$	1/s	2.0	Bayer et al. (2017)	parameter for contraction dynamics: maximum value of B_{rel}
	$\mathcal{S}_{ecc}$	[ ]	2.0	Soest and Bobbert (1993)	ratio of the derivatives of the force-velocity relation at the transition point ($v_{CE} = 0\,\mathrm{m/s}$)
	$\mathcal{F}_{ecc}$	[ ]	1.5	Soest and Bobbert (1993)	factor by which the force can exceed F_{isom} for large eccentric velocities
PEE	$\mathcal{L}_{PEE,0}$	[ ]	0.95	Bayer et al. (2017)	rest length of PEE normalized to optimal length of CE
	ν_{PEE}	[ ]	2.5	Mörl et al. (2012)	exponent of F_{PEE}
	$\mathcal{F}_{PEE}$	[ ]	2.0	Mörl et al. (2012)	force of PEE if l_{CE} is stretched to ΔW_{des}
SDE	D_{SDE}	[ ]	0.3	Mörl et al. (2012)	dimensionless factor to scale $d_{SDE,max}$
	R_{SDE}	[ ]	0.01	Mörl et al. (2012)	minimum value of d_{SDE} (at $F_{MTU} =$ 0N), normalized to $d_{SDE,max}$
SEE	$l_{SEE,0}$	m	0.4	Geyer et al. (2003)	tendon's rest length, alias l_{rest} in Geyer et al. (2003)
	$\Delta U_{SEE,nll}$	[ ]	0.0425	Mörl et al. (2012)	relative stretch at non-linear linear transition
	$\Delta U_{SEE,l}$	[ ]	0.017	Mörl et al. (2012)	relative additional stretch in the linear part providing a force increase of $\Delta F_{SEE,0}$
	$\Delta F_{SEE,0}$	N	0.4 F_{max}	Bayer et al. (2017)	both force at the transition and force increase in the linear part
Hatze	m	1/s	11.3	Kistemaker et al. (2006)	inverse of time constant for the activation dynamics ($1/\tau$, τ defined in TABLE 1)
	c	mol/l	1.37e-4	Kistemaker et al. (2006)	constant for the activation dynamics
	μ	l/mol	5.27e4	Kistemaker et al. (2006)	constant for the activation dynamics
	k	[ ]	2.9	Kistemaker et al. (2006)	constant for the activation dynamics
	q_0	[ ]	0.005	Kistemaker et al. (2006)	resting active state for all activated muscle fibers
	ν	[ ]	3	Kistemaker et al. (2006)	constant for the activation dynamics

References

1. Geyer H, Seyfarth A, and Blickhan R. Positive force feedback in bouncing gaits? Proceedings of the Royal Society of London. Series B: Biological Sciences 2003;270:2173–83. DOI: 10.1098/rspb.2003.2454.
2. Bayer A, Schmitt S, Günther M, and Haeufle D. The influence of biophysical muscle properties on simulating fast human arm movements. Computer methods in biomechanics and biomedical engineering 2017;20:803–21. DOI: 10.1080/10255842.2017.1293663.
3. Kistemaker DA, Van Soest AJ, and Bobbert MF. Is equilibrium point control feasible for fast goal-directed single-joint movements? Journal of Neurophysiology 2006;95:2898–912. DOI: 10.1152/jn.00983.2005.
4. Mörl F, Siebert T, Schmitt S, Blickhan R, and Guenther M. Electro-mechanical delay in Hill-type muscle models. Journal of Mechanics in Medicine and Biology 2012;12:1250085. DOI: 10.1142/s0219519412500856.
5. Soest AJ van and Bobbert MF. The contribution of muscle properties in the control of explosive movements. Biological Cybernetics 1993;69:195–204. DOI: 10.1007/BF00198959.
